# Validity of the Visual Trajectories Questionnaire for Pain

**DOI:** 10.1016/j.jpain.2017.07.011

**Published:** 2017-12

**Authors:** Kate M. Dunn, Paul Campbell, Kelvin P. Jordan

**Affiliations:** Arthritis Research UK Primary Care Centre, Research Institute for Primary Care & Health Sciences, Keele University, United Kingdom

**Keywords:** Pain, measurement, trajectories, questionnaire, validity

## Abstract

Researchers have identified trajectories of pain derived using statistical techniques on longitudinal data. These trajectories have potential to be of use clinically but the repeated data collection required is currently impractical for such situations. Our aim was to investigate the validity of a self-report (Visual Trajectories Questionnaire-Pain) for pain. Analysis included participants from 2 prospective cohorts of people seeking primary health care for back pain (n = 622). A question was developed asking people to classify their pain experience into one of a number of trajectories using visual and word descriptions. Overall 98% of participants completed the question; criterion validity was established by comparing self-report trajectories and trajectories derived using longitudinal latent class analysis, and construct validity was established by comparing responses to the questionnaire against an existing model of back pain stages. As expected variables such as pain intensity and widespreadness, other symptoms, and psychological distress showed an increasing trend of severity across trajectory categories in line with the hypothesized model. In conclusion, the self-report single-item Visual Trajectories Questionnaire-Pain is acceptable to patients and supported by evidence of face, criterion, and construct validity. Further research is needed to investigate the clinical usefulness of the question.

**Perspective:**

This study provides a new questionnaire (Visual Trajectories Questionnaire-Pain) that captures the longitudinal state of a patient's pain experience. The Visual Trajectories Questionnaire-Pain has shown aspects of face, criterion, and construct validity, and has the potential to be clinically useful.

## Highlights

• There is growing clinical/research interest in longitudinal patient pain trajectories.
• Validity of a self-report trajectories question was tested in a back pain population.
• Self-report trajectories were compared with derived longitudinal trajectories.
• Acceptable validity is reported of a new self-report measure of trajectories.

Over the past few years, a number of studies have identified trajectories of back pain.^1^, ^7^, ^14^, ^17^, ^22^ These studies have provided new insights into the course of pain, and indicate that people with back pain can be classified into discrete trajectories with distinct characteristics that have potential clinical usefulness.^2^, ^13^ However, the studies have all used repeated measures collected during prospective longitudinal studies, often with complex analytical techniques, to identify the trajectories and classify the patients. These methods are time-consuming and not always feasible, and indicate that the trajectories currently have limited clinical usefulness, because few clinical situations allow for the collection of longitudinal data to categorize patients.

One solution is to ask patients themselves which trajectory best represents the course of their back pain, and this has been suggested in a recent review of research on back pain trajectories.^13^ Such a question would then allow researchers and clinicians to allocate people with back pain into trajectory groups without having to collect large amounts of data. However, it is not known whether patients can identify their own trajectory, and whether their responses are valid.

There are a number of stages needed to test the validity of such a question. The first element of this is face validity; whether patients can understand the question and assign themselves to a trajectory.^3^ The second component is criterion validity; how well a question compares with an independent external objective criterion or gold standard.^3^, ^18^ For pain trajectories, the external criterion would be the empirical trajectories derived using longitudinal data. The third part would be construct validity, or the extent to which a measure is related to criteria derived from an established theory.^3^, ^18^ One model of pain against which it is useful to make this comparison is the stages of pain model.^20^ This model not only understands chronicity by the temporal experience of pain over time but also incorporates a multidimensional consideration of other types of pain, various bodily complaints, and cognitive and emotional impairments. Evidence shows these conditions are common in those with back pain, are linked to severity, and play a significant role in prognosis.^11^ Testing construct validity using this model would require investigating whether ‘worsening’ trajectories of pain show parallels with different stages of pain and their associated characteristics.

The aim of this study was therefore to investigate the validity of a self-report question (called the Visual Trajectories Questionnaire-Pain, or VTQ-Pain) asking patients to identify the trajectory that best represents their pain experience.

## Methods

This study was nested in 2 cohorts of people seeking primary health care for their back pain (Back Pain Research in North Staffordshire [BaRNS] Study and Beliefs about Back Pain [BeBack] Study). Study participants were consecutive patients visiting their general practitioner about back pain during 2001 and 2002 (BaRNS) or 2004 through 2006 (BeBack); all were invited to take part in a prospective cohort study using questionnaires and followed for up to a year. Further details are published elsewhere.^6^, ^7^, ^9^ The cohorts were followed-up again 7 years (BaRNS) or 5 years (BeBack) later (called the second study period in this report).^4^, ^5^ The second study period consisted of a baseline questionnaire, short monthly questionnaires, and a final questionnaire at 12 months. All phases of both studies were independently approved by the North Staffordshire, South Staffordshire, and North West Cheshire research ethics committees.

A draft question asking patients to classify their back pain experience into a trajectory was developed on the basis of trajectories previously derived through statistical modeling. Four trajectories were developed directly from typical individual trajectories identified within previously published work on the basis of regular reporting of back pain intensity.^7^ The trajectories reported (from 342 consulters) were: persistent mild (n = 122) for patients who had stable low levels of persistent mild pain, recovering (n = 104) for patients who had mild pain to no pain, severe chronic (n = 71) for patients who had permanent high levels of pain, and fluctuating (n = 45) for patients who had pain that moved between mild and high pain over the time period. Three further trajectories were developed using more general information about the course of back pain such as pain that has gradually become worse, having a single episode, and pain that has gradually become better.^16^ These 7 trajectories were thought to capture the range of experience of pain through time and be appropriate for studies in which participants are known to have had a back pain episode within the recall period. An additional item representing no pain was developed for studies in which participants may not have had pain during the recall period. The final question was comprised of 8 pictures of the individual trajectories of pain, with corresponding brief descriptions of each trajectory. The question will be referred to as the VTQ-Pain and was assessed at the 12-month follow-up point of the second study period.

Initial assessments of acceptability and components of face validity were carried out with a small group of patients with experience of musculoskeletal pain—the Research User Group (RUG) at the Research Institute for Primary Care & Health Sciences, Keele University. The RUG has approximately 100 members and many have conditions such as back pain, osteoarthritis, rheumatoid arthritis, ankylosing spondylitis, mental health conditions, and long-term health conditions. The age range is from 33 to 87 years, and there is an even representation from male and female members. RUG members are involved in most aspects of the research process and take part in advisory groups, steering groups, research meetings, coapplicants, and implementation meetings. The group involved in the VTQ-Pain development consisted of 8 members, all with musculoskeletal problems (approximately half with back pain). These RUG members were sent the VTQ-Pain in advance and then invited to a meeting, and asked whether they understood the question, and whether they could suggest any improvements.

After amendments on the basis of RUG feedback (see Results section), the VTQ-Pain was included in the baseline and 12-month second study period questionnaires for the BaRNS and BeBack Study cohorts. The 7-item version was included in the baseline questionnaire, referring to the period since the start of the study (7 years or 5 years previously); the 8-item version (including the no pain trajectory used in this current analysis) was included in the 12-month follow-up questionnaires referring to the previous year. Components of face validity were tested by the views of the RUG feedback, as detailed previously, and also determining the proportion of patients who were able to answer the question in the baseline second study period questionnaires using response/completion rates as an indicator.

Criterion validity was explored by comparing self-report trajectory responses in the 12-month follow-up questionnaire with statistically derived trajectories. These trajectories were derived using longitudinal latent class analysis (LLCA) in both cohorts, using the first 6 months of data from the second study period phase. Monthly reported back pain intensity scores were used to derive trajectories using LLCA; each participant was allocated to a trajectory on the basis of their largest probability. Briefly, pain intensity was measured on a monthly basis using the mean of three 0 to 10 numerical rating scales. These values were trichotomized into no pain (scoring <1), mild-moderate pain, and high pain (score of ≥5) for each month. LLCA was then used to group participants into clusters on the basis of these pain measurements over 6 months. Derived posterior probabilities indicated the probability of a participant belonging to each cluster, and participants were allocated to the cluster for which they had the largest probability of belonging (ie, best match to their pain profile). Cluster-specific probabilities of having each level of pain for each month, considering the membership of that cluster, allowed descriptions of the pain pathways for each cluster. The derived clusters have been shown to have a good fit to the observed patterns.^5^ Full details of how the statistically derived trajectories were developed have been published.^5^ Previous work has shown that trajectory membership is stable over a 1-year period,^7^ and even longer,^5^ so using derived trajectories from the first 6 months of the recalled period is appropriate. Relationships between the VTQ-Pain at the 12-month follow-up and the statistically derived trajectories were hypothesized as in Table 1.

**Table 1:** Hypothesised Relationship Between VTQ-Pain Responses at the 12-Month Follow-up and LLCA-Derived Trajectories

	Visual Trajectory	Hypothesized LLCA Cluster
a)	A single episode with no other major episodes of back pain	No or occasional mild pain
b)	A few episodes of back pain, with mostly pain-free periods in between	No or occasional mild pain
c)	Some back pain most of the time, and a few episodes of severe pain	Persistent mild or fluctuating
d)	Pain that goes up and down all the time, with episodes of severe back pain	Fluctuating or persistent severe
e)	Severe back pain all or nearly all of the time	Persistent severe
f)	Back pain that has got gradually worse	Unclear*
g)	Back pain that has improved gradually	Unclear*
h)	No back pain, or only the odd day with mild pain	No or occasional mild pain

* No specific matches were hypothesized with trajectories f and g.

Construct validity was tested by comparing responses to the VTQ-Pain in the baseline questionnaire of the second study period against constructs supported by the stages of pain model (also assessed at baseline).^20^ In summary, the model proposes stage 0: pain in the back; stage 1: pain radiating elsewhere (below the knee and other parts of the body); stage 2: amplification beyond pain (eg, reduced vitality and occurrence of other symptoms); stage 3: amplification to psychological distress (the occurrence of catastrophizing and/or depression/anxiety), with each stage also including the symptoms of the previous stage. Applying this to the VTQ-Pain responses, we would expect that people self-reporting trajectories with no pain most of the time would be closest to stage 0, those with trajectories indicating repeated pain episodes but no pain a lot of the time would have characteristics of stage 1, those with constant mild pain would be closest to stage 2 and those with constant severe or fluctuating pain would be closest to stage 3.

Pain in the back was represented by pain intensity at baseline using the mean of three 0 to 10 numerical rating scales.^8^ Pain radiating elsewhere was measured as the proportion of patients with pain spreading below the knee, and the proportion with pain elsewhere in the body (shoulder, arm, neck, or head). Amplification beyond pain was measured using the vitality subscale of the short form 12 (SF12) in the BaRNS study only,^23^ somatic symptoms from the 15-item Patient Health Questionnaire (scored from 0 [not bothered with any symptoms] to 30 [bothered a lot with all 15 symptoms]),^15^ insomnia (proportion reporting having trouble falling or staying asleep, waking up several times at night, or waking up feeling tired on most nights),^12^ and disability (Roland-Morris Disability Questionnaire).^19^, ^21^ Amplification to psychological distress was measured using a measure of catastrophizing (full 5-item catastrophizing subscale of the Coping Strategies Questionnaire 24 for the BaRNS study,^10^ and a single-item dichotomous catastrophizing item from the same scale in the BeBack study), and the anxiety and depressive symptoms subscales of the Hospital Anxiety and Depression Scale (scored from 0 to 21, with higher scores indicating more severe symptoms).^24^ We determined using linear/logistic regression the amount of variance explained (eg, R^2^) by the VTQ-Pain and by the LLCA trajectories for each of the construct validity variables.

## Results

**Figure 1:**
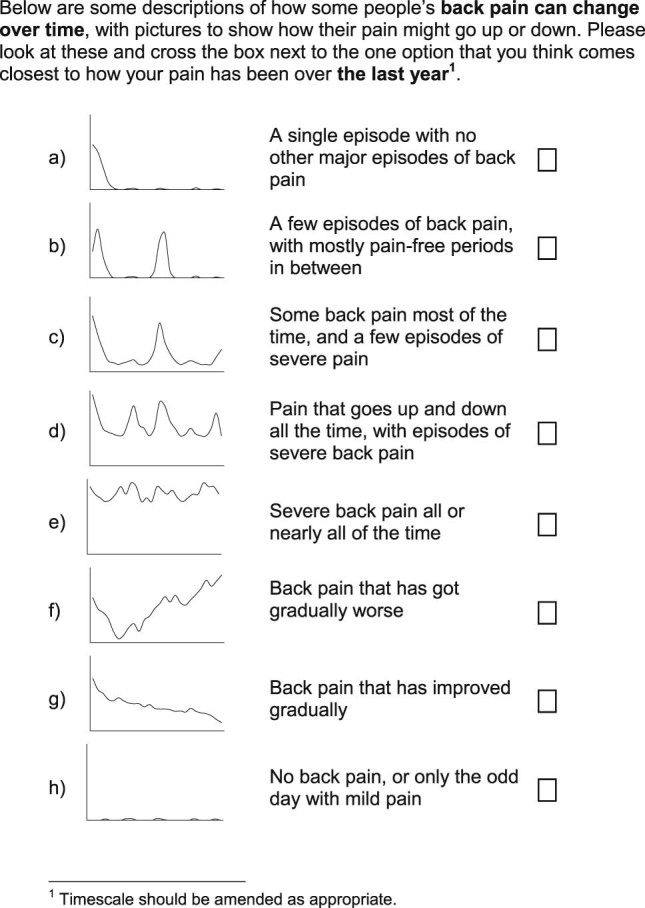
VTQ-Pain.

The patients in the RUG group reported that the VTQ-Pain was easy to understand, they did not report any difficulty in understanding the axes, there was no mention of additional trajectories, and they would be able to complete it. They suggested a minor amendment to the formatting of the trajectory pictures that they believed would make them more easily understood (original version had the area under the line shaded, the RUG asked for this to be removed). The final VTQ-Pain is presented in Fig 1.

### Face Validity

In the second study period baseline questionnaires, 98% of respondents were able to answer and complete the VTQ-Pain (202 of 208 in BaRNS and 420 of 429 in BeBack). Similar response frequencies were found in the 12-month follow-up. Frequencies of response to the individual trajectories at baseline are shown in Table 2. These indicate that the proportion of people selecting each trajectory was very similar between the 2 cohorts. The most common trajectory selected (40%) indicated that a large proportion of responders experienced “A few episodes of back pain, with mostly pain-free periods in between.” The next most common trajectory (24% of responders) was “Some back pain most of the time, and a few episodes of severe pain.”

**Table 2:** Response to the VTQ-Pain in the Long-Term Follow-up Baseline Questionnaires

		BaRNS Study		BeBack Study		Total	
		n	%	n	%	n	%
a)	A single episode with no other major episodes of back pain	14	6.9	33	7.9	47	7.6
b)	A few episodes of back pain, with mostly pain-free periods in between	79	39.1	172	41.0	251	40.4
c)	Some back pain most of the time, and a few episodes of severe pain	47	23.3	102	24.3	149	24.0
d)	Pain that goes up and down all the time, with episodes of severe back pain	28	13.9	69	16.4	97	15.6
e)	Severe back pain all or nearly all of the time	8	4.0	15	3.6	23	3.7
f)	Back pain that has got gradually worse	12	5.9	17	4.0	29	4.7
g)	Back pain that has improved gradually	14	6.9	12	2.9	26	4.2
	Total	202		420		622	

### Criterion Validity

The self-reported visual trajectory responses given in the 12-month questionnaire from the second study period of the studies were compared with trajectories derived using LLCA for the 2 cohorts (n = 373) in Table 3. These indicate that the observed relationships between self-reported VTQ-Pain responses and the derived trajectories were broadly in line with the hypothesized relationships (Table 1). For example, 73% of those reporting a visual trajectory of “A single episode with no other major episodes of back pain,” and 86% of those reporting “No back pain, or only the odd day with mild pain” were observed to have a statistically derived trajectory of no or occasional mild pain. Similarly, 77% of those reporting “Severe back pain all or nearly all of the time” had a statistically derived trajectory of persistent severe pain. However, there were some differences between hypothesized and observed relationships; for example, only 36% of those reporting “A few episodes of back pain, with mostly pain-free periods in between” were classified as having no or occasional pain within the LLCA trajectories, with the most (56%) classified within the persistent mild pain LLCA trajectory. This may have been driven by increased frequency (episodes) and in this case persistent may also include some with pain-free episodes, which are less frequent. Comparison of the “Some back pain most of the time, and a few episodes of severe pain” with the “Pain that goes up and down all the time, with episodes of severe pain” categories shows the former have most (62%) of respondents classified within the persistent mild pain LLCA trajectory, whereas the latter had most (62%) within the persistent severe pain LLCA trajectory. With regard to the 2 categories that had no direct LLCA trajectory equivalent, those who described themselves as “Back pain that has got gradually worse” show a spread of representation across the LLCA trajectories, with most (44%) in the persistent severe pain trajectory, and those who describe themselves as “Back pain that has improved gradually” are mainly concentrated in the persistent mild pain LLCA trajectory.

**Table 3:** Comparison of the VTQ-Pain Responses at Long-Term 12-Month Follow-up With the 4 Trajectories Derived From LLCA (BaRNS and BeBack Combined)

		No or Occasional Mild Pain	Persistent Mild Pain	Fluctuating	Persistent Severe Pain	Total
a)	A single episode with no other major episodes of back pain	8 (73)	3 (27)	0 (0)	0 (0)	11
b)	A few episodes of back pain, with mostly pain-free periods in between	42 (36)	66 (56)	5 (4)	4 (3)	117
c)	Some back pain most of the time, and a few episodes of severe pain	3 (4)	51 (62)	13 (16)	15 (18)	82
d)	Pain that goes up and down all the time, with episodes of severe back pain	0 (0)	21 (31)	5 (7)	42 (62)	68
e)	Severe back pain all or nearly all of the time	0 (0)	3 (23)	0 (0)	10 (77)	13
f)	Back pain that has got gradually worse	2 (22)	3 (33)	0 (0)	4 (44)	9
g)	Back pain that has improved gradually	2 (14)	12 (86)	0 (0)	0 (0)	14
h)	No back pain, or only the odd day with mild pain	51 (86)	8 (14)	0 (0)	0 (0)	59
	Total	108	167	23	75	373

NOTE. Data are presented as n, or n (%).

### Construct Validity

All variables showed an increasing trend of severity across trajectory categories from: a (single episode) to e (persistent severe back pain), meaning that patients with less frequent and less severe pain have better health than patients with more frequent and severe pain (see Table 4, Table 4). This is consistent with the stages of pain model. The VTQ-Pain trajectories with no pain most of the time (categories a and b) are closest to stage 0, displaying no or mild pain (mean pain intensity <2) and <10% overall reporting radiating pain in the leg. People with constant mild pain (category c) appear to have characteristics of stage 1, with up to 40% reporting pain radiating down the leg and approximately 80% reporting pain elsewhere in the body. Respondents with fluctuating pain (category d) had higher levels of somatic symptoms and insomnia than the respondents with milder trajectories, indicating stage 2, and people with persistent severe pain (category e) have the highest levels of depression, indicating stage 3. Category f (worsening pain) showed characteristics similar to stage 3, and category g (improving pain) showed characteristics similar to stage 0. There was a generally similar level of variance explained by the VTQ-Pain response and by the LLCA trajectories, for each of the construct validity variables, although the LLCA trajectories explained more of the variance for depression (see Table 5).

**Table 4:** Construct Validity—VTQ-Pain Responses Against Constructs Derived From the Stages of Pain Model

		Back Pain Intensity	Pain Radiates to Below the Knee	Pain Elsewhere	Vitality	Symptoms	Insomnia	Disability	Catastrophizing	Depression	Anxiety
BaRNS 7-year follow-up baseline data											
a)	A single episode with no other major episodes of back pain	.19 (−.03 to .41)	0%	14%	3.57 (3.08–4.06)	2.79 (1.06–4.51)	46%	.29 (−.19 to .76)	1.13 (−.57 to 2.82)	2.36 (.99–3.73)	4.36 (2.79–5.95)
b)	A few episodes of back pain, with mostly pain-free periods in between	1.17 (.89–1.45)	14%	64%	3.27 (3.07–3.46)	3.94 (3.24–4.63)	31%	2.16 (1.61–2.72)	.74 (.44–1.04)	3.46 (2.6–4.32)	5.46 (4.58–6.34)
c)	Some back pain most of the time, and a few episodes of severe pain	3.57 (3.01–4.13)	40%	81%	2.68 (2.43–2.93)	6.09 (4.85–7.34)	57%	7.00 (5.46–8.54)	1.58 (.96–2.2)	6.00 (4.87–7.13)	8.00 (6.81–9.19)
d)	Pain that goes up and down all the time, with episodes of severe back pain	5.62 (4.73–6.51)	36%	89%	2.50 (2.14–2.86)	8.04 (6.37–9.71)	64%	11.32 (9.34–13.3)	2.96 (2.19–3.73)	6.71 (5.3–8.13)	8.14 (6.88–9.41)
e)	Severe back pain all or nearly all of the time	6.57 (4.09–9.06)	86%	86%	1.75 (1.16–2.34)	8.14 (3.63–12.66)	75%	14.63 (8.20–21.05)	4.00 (2.21–5.79)	7.75 (3.54–11.96)	9.63 (4.51–14.74)
f)	Back pain that has got gradually worse	7.30 (5.40–9.20)	64%	90%	1.73 (1.12–2.33)	9.30 (3.23–15.37)	92%	12.83 (8.16–17.51)	4.56 (3.16–5.95)	7.83 (5.38–10.28)	10.17 (7.72–12.62)
g)	Back pain that has improved gradually	1.07 (.15–2.00)	36%	71%	3.50 (2.83–4.17)	3.43 (1.42–5.44)	14%	2.57 (-.39–5.53)	1.75 (.09–3.41)	3.29 (1.24–5.33)	3.86 (2.02–5.69)

		Back Pain Intensity	Pain Radiates to Below the Knee	Pain Elsewhere	Symptoms	Sleep Problems	Disability	Catastrophizing	Depression	Anxiety
BeBack 5-year follow-up baseline data										
a)	A single episode with no other major episodes of back pain	.12 (.03–.23)	12%	27%	3.68 (2.68–4.74)	19%	.33 (.07–.59)	0%	3.15 (2.27–4.06)	5.50 (4.06–6.94)
b)	A few episodes of back pain, with mostly pain-free periods in between	1.33 (1.07–1.58)	22%	56%	5.69 (4.86–6.54)	36%	2.83 (2.23–3.42)	1%	3.94 (3.44–4.47)	6.71 (6.05–7.36)
c)	Some back pain most of the time, and a few episodes of severe pain	3.37 (3.00–3.72)	29%	81%	9.00 (7.64–10.43)	55%	6.01 (4.99–7.03)	17%	4.92 (4.23–5.55)	7.75 (6.85–8.64)
d)	Pain that goes up and down all the time, with episodes of severe back pain	4.86 (4.36–5.38)	48%	80%	10.22 (8.81–11.70)	80%	11.33 (9.96–12.70)	36%	7.06 (6.15–8.09)	8.56 (7.52–9.60)
e)	Severe back pain all or nearly all of the time	7.64 (6.84–8.40)	73%	80%	12.83 (7.34–18.33)	79%	16.27 (14.84–17.69)	73%	8.00 (5.86–10.07)	10.62 (7.81–13.42)
f)	Back pain that has got gradually worse	6.10 (5.08–7.00)	41%	82%	8.90 (6.10–11.70)	59%	10.94 (7.38–14.50)	47%	7.18 (5.12–9.53)	9.24 (7.09–11.38)
g)	Back pain that has improved gradually	1.17 (.61–1.80)	25%	50%	5.50 (1.50–9.50)	17%	2.75 (.53–4.97)	0%	2.50 (1.33–3.67)	5.08 (3.28–6.89)

NOTE. Data are mean with 95% confidence interval or proportion. For all variables, increasing values indicate increasing severity, except for vitality, for which the opposite is true.

**Table 5:** Comparison of VTQ-Pain and LLCA for Construct Variable Variance (R^2^)

Construct	BaRNS 7-Year Follow-up Baseline Data		BeBack 5-Year Follow-up Baseline Data	
	LLCA R^2^	VTQ-Pain R^2^	LLCA R^2^	VTQ-Pain R^2^
Pain Intensity	.243	.192	.202	.195
Pain radiates to below the knee*	.216	.265	.195	.165
Pain elsewhere*	.185	.239	.201	.159
Vitality*	.202	.350		
Symptoms	.091	.117	.103	.111
Insomnia/sleep problems*	.255	.208	.255	.198
Disability	.158	.164	.207	.145
Catastrophizing*	.187	.148	.154	.182
Anxiety*	.184	.220	.066	.067
Depression*	.279	.070	.306	.168

* Nagelkerke values.

## Discussion

We have shown that a new single-item VTQ-Pain, which asks people to categorize themselves into trajectories of pain, is supported by evidence of face, criterion, and construct validity in 2 independent cohorts of primary care back pain consulters. The question is acceptable to patients, and people selecting different response categories are also different in other ways including their statistically derived trajectories of pain, pain radiation and spread, and amplification to other symptoms and psychological distress.

There is support for concordance between the reported trajectories and the LLCA clusters. Most respondents who described their trajectory as having no back pain, improving back pain, or only having a single episode fell within the no or occasional mild pain LLCA trajectory (and none were found in the fluctuating or severe pain LLCA trajectories), whereas those who chose severe pain all the time, pain that goes up and down with severe episodes, or back pain that has gradually worsened, were predominantly in the persistent severe pain LLCA trajectory. An assignment of variables broadly in line with the stages of pain model was shown, but there was not always a clear distinction between the stages and “gray” areas will exist using such categorizations. For example, there were gradually increasing mean levels of anxiety as the visual trajectory severity increased, rather than a sudden leap of scores from the other trajectories to the trajectory representing severe pain all or nearly all of the time. Evidence from previous work also shows that rather than a set of stages through which people progress over time,^5^ the categories are more likely to reflect different groups of people who remain with similar characteristics over time (ie, more like phenotypes than transitional phases) with overlap between these phenotypes.

### Strengths and Limitations

This study has the strength of testing the VTQ-Pain in 2 independent cohorts of primary care back pain consulters. However, because of the nature of identification and retention of participants included in this study, we cannot give estimates of the prevalence of the visual trajectories. There may be different proportions of people identifying with the response categories in different studies and settings, and this remains to be tested. Testing criterion validity against the reference standard of statistically derived trajectories is a strength. However, agreement between self-report and statistically derived trajectories was limited, possibly reflecting bias in recall of trajectories, compared with trajectories derived using longitudinal data. There were also limited numbers for the analysis with LLCA-derived trajectories, and although previous work has shown that people providing data for longitudinal analyses are broadly similar to the whole sample,^5^ the possibility for bias remains. Another strength is the wide range of variables included in the testing of construct validity for this question, within 2 different data sets, and all showed validity (patterns in the expected directions) against the existing construct (stages of pain model) as well as similarity in extent of variance explained by the LLCA and self-report trajectories. This study also has a number of limitations. Although this study carried out a review of the measure's acceptability/readability by the RUG and the response rate of measure completion in the 2 cohorts was 98%, suggesting the question was acceptable and relevant to responders, there was no inclusion of a “read aloud” session with the RUG or participants to assess the cognitive process of interpretation of the question. In addition, there was no option for respondents who did not recognize any of the patterns (eg, I do not recognize any of the patterns of pain over time), therefore the study may have missed some information to improve or refine the measure and more rigorous testing of face validity is required. Reliability of the measure was not assessed (test-retest). It is also possible that using a shorter recall time (eg, over the previous month) would provide a better comparison with LLCA trajectory clusters than recall over 12 months. Further work is needed to establish the optimum and nonoptimum range of recall period for which the VTQ-Pain can be used.

LLCA did not identify systematically increasing or decreasing trajectories of change. Only 6% of the population self-reported such patterns (group f—back pain that has got gradually worse, group g—back pain that has improved gradually). This may be a reflection of this population (people with long-term back pain). Inspection of the baseline levels of pain intensity for these groups show high pain levels for group f (>7) and low levels for group g (<2) and this may reflect the relative stability of pain within this cohort (2 long-term back pain cohorts) with little room to reflect change in the 12-month period. It may be that the relative frequency of the trajectory groups, including those that capture change over time, may well be different for different populations (for example, if measuring from time of first consultation for back pain). Kongsted et al^14^ recently reported on an inception cohort of consulters (ie, first time of consultation for low back pain) with LLCA trajectories derived from weekly measurements over a 12-month period. They report, using multiple models, 5 to 8 subgroups, with only a small percentage grouped as changing (improvement, worsening, fluctuating) whereas most (>60%) were in stable clusters. This highlights the stability of trajectories, even in a population in whom more change would be expected and this current study showed participants reporting visual trajectory patterns a to e or h; 63.4% had an expected LLCA trajectory. Furthermore, although there is broad agreement between the participants' chosen trajectory and the LLCA clusters it is not absolute and variation will exist in the interpretation of the trajectories for each person, for example, people who have chosen the same trajectory may have chosen differently if asked prospectively, or asked at repeated points over time. The VTQ-Pain has only been tested in those who have reported back pain (most of whom would have low back pain) and there may be different responses given for different pain conditions. However, the measurement of trajectories in this current study is on the basis of pain intensity, which can be considered a universal measure across varied pain conditions.

### Clinical Relevance

The VTQ-Pain has potential clinical usefulness, because it is simple for patients to answer, and provides relevant information about other characteristics of the patient. In addition, it allows the measurement of trajectories over time without the need to collect data longitudinally. Although there are, as yet, no treatments designed to be matched to different pain trajectories, the characteristics of the patients in the different trajectories do present potential targets for intervention. For example, patients who are mostly pain free may benefit from simple advice and reassurance, whereas patients with mild pain most of the time may require more management of pain elsewhere and other symptoms, and people with constant higher levels of pain may require interventions targeting psychological aspects of their health as well as their pain and other symptoms. Future research may provide more information about which treatments could be best matched to patients in the different trajectory groups. Furthermore, the visual trajectories question may have the potential to be used as an outcome measure, for example, to illustrate change in course after an intervention, however, further research would be needed to test such a measure within this context (eg, testing of responsiveness).

## Conclusions

We have developed an acceptable single-item question on visual trajectories of pain, with evidence of validity, and potential usefulness in research and clinical practice.
